# Dietary Intake and Obesity among Filipino Americans in New Jersey

**DOI:** 10.1155/2018/6719861

**Published:** 2018-09-16

**Authors:** Persephone Vargas

**Affiliations:** William Paterson University, 300 Pompton Rd., Wayne, NJ 07470, USA

## Abstract

The prevalence of obesity is a public concern and is linked to chronic diseases. Filipino Americans have a high prevalence rate of hypertension and diabetes. This study investigated the dietary intake of first-generation Filipino Americans (*n*=210). In addition, it provides a comparison of the obesity rates using the International guideline and the WHO Asian recommendation. The dietary intake included caloric, carbohydrate, and fat intake and was determined using the Block Brief Food Frequency Questionnaire. The anthropometric measurements included actual height, weight, and waist measurements. The body mass index (BMI) and waist circumference were categorized using the International guideline and the WHO Asian recommendation to determine obesity. The caloric and carbohydrate intake were normal; however, fat intake was increased. The BMI and waist circumference showed substantial difference when using the International and Asian guidelines to determine obesity. The results highlight the increased health risks of Filipino American immigrants including a high dietary fat intake and an increased obesity rate.

## 1. Introduction

In the United States, obesity is a public health concern with 34.9% of the adult population considered obese [1]. Overweight and obesity are major risk factors of chronic diseases such as hypertension (HTN), diabetes (DM), and cardiovascular disease (CVD) [2]. Obesity increases the risk for all-cause CVD mortality [2]. It greatly impacts healthcare economy with the medical care cost of obesity estimated at $147 billion [2].

The current U.S. guideline uses the WHO International classification to define obesity as a body mass index (BMI) of ≥30 kg/m^2^ [3]. Using the International BMI measurement for obesity, Asian Americans have a low obesity rate (10.8%) compared to all other races [1]. However, despite having a low obesity rate, Asians have a higher percentage of body fat compared to other races of the same age, sex, and BMI [4]. This results in Asians having a higher morbidity and mortality even with a lower BMI.

In 2004, the World Health Organization (WHO) expert consultation proposed lowering the BMI cut-off points for Asians [4]. The WHO Asian BMI recommendation define overweight as a BMI ≥ 23 kg/m^2^ to <27.5 kg/m^2^ and obesity as BMI ≥ 27.5 kg/m^2^ [4, 5]. In studies using the WHO Asian BMI cut-off, a significant increase in the overweight and obesity rates among the Asian American population was found [6, 7]. A study using the WHO Asian BMI cut-off points showed that the Filipino American overweight/obesity rate (78.6%) was higher than non-Hispanic Whites (53.8%), African Americans (64.9%), and Hispanics (69.7%) [8]. In another study, the obesity rate for Filipino American men was 34.5%, which was similar to the national obesity rate (34.9%) [9].

Abdominal obesity is an indicator of visceral adiposity and is strongly associated with CVD and diabetes [10]. Waist circumference is a good predictor of abdominal fat and is related to increased all-cause mortality [10]. In the U.S., the waist circumference cut-off points follow the WHO waist circumference cut-off recommendation which considers substantially increased risk for waist circumferences >35 inches in women and >40 inches in men [11]. However, compared to Caucasians, Asians have a higher visceral fat [10]. This increased health risk despite lower waist circumference cut-off points warranted alternative cut-off points for the Asian population [10]. The recommended WHO Asian waist circumference cut-off points for increased health risks are >31.5 inches in women and >35.5 inches in men [9].

Diet plays an important role in obesity [2]. Excess calorie intake along with overconsumption of specific nutrients and foods such as saturated fat, added sugars, high sodium food, and refined grains contribute to obesity [2]. Studies on Filipino American diet have shown a change in the dietary intake of Filipino immigrants. This is consistent with dietary acculturation, the changes that occur in an immigrant's diet as they adopt to the dietary practices of the host country [12]. As early as 1975, a study on Filipino American diet had already shown an increase in caloric intake, with twice as many from protein (19%), three times as many from fat intake (41%), and half as many from carbohydrate intake (40%) [13]. The most common dietary change noted among Filipino Americans is the increased consumption of meat and dairy [14]. Dietary acculturation is the change that occurs in an immigrant's diet as they adopt to the dietary practices of the host country [14]. Among Filipino Americans, Western dietary acculturation has been found to be correlated with a higher sugar, fat, and caloric intake [15, 16]. Western dietary acculturation was also correlated with increased BMI and waist circumference [15, 16].

There is an estimated 3.9 million Filipinos in the United States, making them the second largest Asian subgroup [17]. Filipinos are also the second largest immigrant group, with 1.9 million (66%) born outside of the U.S. [17]. New Jersey (NJ) ranks among the top five states in the U.S. with the most Filipino immigrants, with a Filipino population of 120,171 of which 75% are foreign-born [17]. Filipino Americans have an ideal sociodemographic profile with majority having a college degree, a significant number working as healthcare professionals, and a higher mean income [17].

Despite the ideal sociodemographic profile, the Filipino Americans suffer a concerning health disparity. Filipino Americans have significantly increased rates of chronic diseases such as cardiovascular disease (CVD), hypertension (HTN), diabetes (DM), and other CVD risk factors [18–21]. Studies have consistently reported the HTN prevalence rate in Filipino Americans as 53–59.9% [18, 21], which is similar to the African Americans HTN prevalence rate. An analysis of the California Health Interview Survey revealed that the Filipino Americans had the highest BMI among all Asian American subgroups [20]. In addition, Filipino Americans had the second highest prevalence rate of diabetes, 15.8%, second only to the Native Americans [20]. In comparison, the prevalence rate in the Philippines for diabetes is 5.9% [22], HTN is 22.3% [22], and obesity is 5.2% [23].

This article aims to present the dietary intake, including daily caloric, carbohydrate, and fat intake of Filipino Americans in New Jersey. This article also presents additional analysis of data that was previously reported in the *International Journal of Environmental Research and Public Health* [16]. The initial article presented the acculturation level and dietary pattern among first-generation Filipino American immigrants and the relationship among these variables with dietary-related health indicators. This current article provides a secondary analysis of the participants' BMI and waist circumference using the WHO Asian recommendation in comparison with the standard U.S. guidelines to determine obesity.

## 2. Methods

### 2.1. Study Design and Participants

A descriptive cross-sectional study was conducted among first-generation Filipino Americans in New Jersey (NJ). The study included a convenience sample of adult Filipino Americans over the age of 18. All participants had to speak and read English. Pregnant women, those with medical condition that required a prescribed therapeutic diet, and those who had less than one year of residency in the U.S. were excluded from the study [16].

### 2.2. Procedures and Measures

Approval was obtained from the William Paterson University Institutional Review Board on May 20, 2014. Participants were recruited from Filipino organizations, churches, and festivals. Snacks were provided during data collection in the churches and organizations. A $10 target gift card was provided as participation incentive during the Filipino festivals. All participants were asked to sign an informed consent prior to participation. All questionnaires were completed by the participants.

The participants' sociodemographic data and dietary intake were collected using pen and paper survey. Sociodemographic information included sex, age, immigration information, education, occupation, and income. To ensure anonymity, no identifying information was obtained.

The Block Brief Food Frequency Questionnaire (BFFQ) was used to estimate the participants' dietary intake. The BFFQ is a self-administered, 70-item questionnaire, developed from the National Health and Nutrition Examination Survey (NHANES) III dietary recall data [24]. It estimates the dietary intake of the most common foods. The completed questionnaires were sent to NutritionQuest for analysis. The nutritional variables for this study are daily caloric intake, carbohydrate intake, and fat intake.

Once the participants completed the questionnaires, anthropometric measurements were obtained including actual weight, height, and waist circumference. All anthropometric measurements were done by the author to ensure consistency.

The participants' height was measured using a portable stadiometer, to the nearest 0.1 inch (in.). Weight was measured using a digital weighing scale, to the nearest 0.1 pound (lb). All participants were measured with only one layer of clothing and were asked to remove their shoes. The participants' height and weight were used to calculate their BMI using the National Heart, Lung and Blood Institute (NHLBI) calculator [25].

The participants' waist circumference was measured using a nonstretchable measuring tape at the level of the umbilicus with the tape measure parallel to the floor. The measurement was obtained at the end of normal exhalation and rounded off to the nearest 0.5 inch.

The participants' BMI and waist circumference measurements were evaluated using both the standard guidelines and the WHO Asian recommendation. The International guideline defines overweight as having a BMI ≥ 25 to ≤30 and obesity as having a BMI ≥ 30 [2]. Using the WHO Asian BMI cut-off, the categories are normal (BMI 18.5 to <23), overweight (BMI ≥ 23 to <27.5), and obese (BMI ≥ 27.5) [3, 4]. For the waist circumference, the International guideline cut-off points for increased health risks are >35 inches in women and >40 inches in men [10]. Using the WHO Asian cut-off, the waist circumference cut-off points for increased health risks are >31.5 inches in women and >35.5 inches in men [10].

### 2.3. Analysis of Data

All data collected were entered and computed using SPSS 21. Descriptive statistics were used to summarize sociodemographic data, nutritional intake, BMI, and waist circumference. The BMI categories and the waist circumference categories were calculated for both the International guideline and the WHO Asian recommendation. Pearson's correlation was used to investigate the relationship between sociodemographic factors, dietary intake, and anthropometric measurements. A value of *p* < 0.05 was considered statistically significant.

## 3. Results

A total of 210 Filipino Americans were recruited to participate in the study. The sociodemographic information, as previously reported [16], included age, sex, marital status, education, occupation, and immigration data. The mean age of participants was 46.6 (SD = 13.6) ranging from 18 to 74 years. One hundred thirty-four (63.8%) of the respondents were female and 76 (36.2%) were male. Majority of the respondents were married (74.3%), had a college degree or higher (81.9%), and were currently employed (76.7%). The most common occupation was in health care with 38.1% working as a healthcare professional. Most participants had an individual income of $50,000 or above (55.7%), with the largest percentage of participants earning $75,000–99,999 (21.9%).

Immigration information included the length of residency in the U.S. and age of immigration. The length of residency in the U.S. ranged from 1 to 49 years with a mean of 17.6 years (SD = 9.2). The age upon immigration ranged from 1 to 62 years old, with a mean of 28.62 years (SD = 11.8) [16].

Table 1 presents the dietary intake of the participants. The mean daily caloric intake was 1513.2 kcal (SD = 723.3), mean fat intake was 35.2% (SD = 7.7), and mean carbohydrate intake was 50.2% (SD = 9.1). When analyzed by gender, the mean caloric intake for men is 1,648.6 kcal (SD = 739.1), fat intake was 34.6% (SD = 7.6), and carbohydrate intake was 50.8% (SD = 9.3) [21]. The mean caloric intake for the women was 1,436.5 kcal (SD = 705.4), fat intake was 35.5% (7.7), and carbohydrates was 50.1% (SD = 9).

The relationship between dietary intake (caloric intake, fat intake, and carbohydrate intake), age, immigration status (length of residency and immigration age), and sociodemographic data (gender, marital status, education, occupation, and income) was investigated. There were no significant relationships found between dietary intake and sociodemographic data. The correlations between dietary intake, age, length of residency in the U.S., and immigration age are presented in Table 2. Age was found to be significantly correlated with carbohydrate intake (*r*=0.212, *p* < 0.05): those who were older had a higher carbohydrate intake. A negative correlation was found between immigration age and fat intake (*r*=−1.158, *p* < 0.05): those who migrated at an older age had lower fat intake. A significant correlation was also found between immigration age and carbohydrate intake (*r*=0.2458, *p* < 0.001): those who migrated at an older age had a higher carbohydrate intake.

The participant's mean BMI was 25.4 (SD = 3.6) [16]. When analyzed by gender, the male participants' mean BMI was 26.7 (SD = 4.1) and the female participants' mean BMI was 24.7 (SD = 3.2) [16]. The participants' BMI was categorized as normal, overweight, and obese using both the International guideline and the WHO Asian BMI recommendation.  Table 3 shows a comparison of the BMI categories between the International guideline and the WHO Asian recommended BMI cut-off. In men, the obesity rate doubled while in women there was more than a threefold increase when the WHO Asian BMI was used to determine obesity.

The male mean waist circumference was 35.9 inches (SD = 4.8). The female mean waist circumference was 32.3 inches (SD = 3.7) [16].  Table 4 shows a comparison of the waist circumference categories between the International guideline and the WHO Asian recommendation. In both men and women, those classified with increased risk more than doubled when using the WHO Asian waist circumference cut-off.

The relationship between anthropometric measurements and sociodemographic factors was investigated. There were no statistically significant differences noted in BMI when compared by sex, marital status, education, income, or occupation. An analysis of the correlation between age and immigration status showed a significant correlation between age and waist circumference (*r*=0.282, *p* < 0.001): those who were older had an increased waist circumference and WHR. There was also a significant correlation between immigration age and waist circumference (*r*=0.275, *p* < 0.001): those who came to the U.S. at an older age had an increased waist circumference.

## 4. Discussion

This study highlights the significant difference between the International BMI guideline and the WHO Asian BMI recommendation to determine obesity in the Filipino American population. When using the WHO Asian BMI recommendation, there was a significant increase in the overweight and obesity rates of the participants. The number of participants with an increased waist circumference indicating increased health risks was also significantly higher when using the WHO Asian waist circumference cut-off points.

The results of this study indicate an overwhelming underestimation of obesity and abdominal obesity in the Filipino Americans when using the International BMI and waist circumference guidelines. In total, the obesity rate increased from 9.5% to 25.2% when using the WHO Asian BMI cut-off. The obesity rate in men was doubled (15.8% to 31.6%), and the obesity rate in women increased more than threefold (6% to 21.6%) when using the WHO Asian BMI cut-off points [21]. For the waist circumference, half of the participants exceeded the WHO Asian waist circumference cut-off points, indicating increased health risks.

Ursua et al. [21] had similar findings with the Filipino Americans in the New York area. The study also used both the International guideline and WHO Asian recommendation to categorize BMI. Using the International guideline, 48.4% of the participants had a normal BMI and 51.6% were overweight or obese. However, using the WHO Asian BMI cut-off, 25.5% of the participants had a normal BMI and 74.5% were overweight or obese. The study did not categorize BMI according to gender and did not use waist circumference.

Other studies have used both the International guideline and the WHO Asian recommendation to categorize the BMI of Filipino American immigrants. In using the International BMI guideline, the other studies have shown that anywhere from 44 to 66% of Filipino Americans are overweight or obese. However, these numbers become more concerning when categorized using the WHO Asian recommendation, with 60–80% in the overweight or obese category [6, 8, 26].

Filipino Americans have been found to have significantly higher visceral adiposity compared to Caucasians and African Americans [27]. Increased BMI and waist circumference have been linked to increased risk for cardiovascular disease, diabetes, other chronic diseases, and mortality. Asians have a higher risk for metabolic disease, diabetes, and cardiovascular disease compared to Caucasians with BMI (25 kg/m^2^) and waist circumference (40 inches in men, 35 inches in women) below the existing cut-off points [4, 10]. Thus, it is important to use the appropriate guideline in determining obesity for this population.

Another important finding of the study is the increased fat intake among the participants. The mean daily caloric intake (women = 1,436.5 kcal, men = 1,648.6 kcal) in this study was lower than the recommended dietary guidelines (women = 1,600–2,400 kcal, men = 2,000–3,000 kcal) [28], and the carbohydrate intake was within the normal range (50.2%) of the dietary recommendation (45–65%) [28]. However, the fat intake (35.2%) was above the dietary recommendations (20–35%) [28]. It is important to note that more than half (52%) of the participants had a fat intake greater than the dietary recommendation.

The finding of increased mean fat intake in this study is consistent with the other studies on Filipino American diet. The most common dietary change that occurs when Filipinos immigrate to the U.S. is the increased consumption of meat and fat [12, 14–16]. The studies indicated that the participants consumed more beef, pork, chicken, dairy products, and fast foods. This dietary change is mostly due to the availability and affordability of these types of food [14]. In comparison, the average diet of Filipinos in the Philippines, according to the Philippine Food and Nutrition Research Institute, consists of rice, fish, vegetables, chicken, and fruits [29]. Rice represents 42% of the total average daily intake, vegetables 13%, fish 12%, fruits 9%, and meat and poultry 6% [29]. The increase in fat intake among Filipino Americans is a health concern since it is a risk factor for several chronic diseases including CVD and DM.

Rice remains the staple food of Filipino American immigrants and the major source of carbohydrates [13, 14, 30]. In addition to rice, Filipino Americans also eat more bakery products [14]. The study also showed an increase in carbohydrate intake among the older participants and those who migrated at an older age, and a decrease in fat intake in those who migrated to the U.S. at an older age. It is plausible that the older participants and those who migrated at an older age are used to having rice with each meal, which is traditional [14]. They may have not have adapted to the Westernization of diet which can lead to a decrease in rice and carbohydrate intake and increase in fat intake [15, 16].

There is a concern that the use of the food frequency questionnaire may lead to underestimation of dietary intake since it does not include commonly eaten traditional food or reflect dietary practices [30]. Quantifying food intake frequency and amount becomes a challenge because Filipino Americans tend to serve meals family-style. The participants have the tendency to base their answers on what their family ate and felt guilty when they thought about how much they ate [30]. This may explain the low caloric intake finding in the study. To improve the accuracy of dietary intake measurement among Filipino immigrants, an FFQ to reflect Filipino dietary practices should be considered.

It is important to note that the sociodemographic factors did not show a statistically significant difference in the dietary intake, BMI, and waist circumference. This result is similar to the findings by Serafica et al. [15]. The Filipino American immigrants have an ideal sociodemographic profile: majority of them have a college degree, work in the healthcare field, and have a higher income [17]. However, these sociodemographic factors did not improve the dietary intake or the anthropometric findings in this population. These findings show that all Filipino Americans regardless of socioeconomic status have increased health risk factors.

To the best of the author's knowledge, this is the first study that investigated the dietary intake of Filipino Americans in the Northeast area. Only one other study examined the BMI of Filipino American in the NY/NJ area [21]. The study utilizes actual anthropometric measurements, instead of self-reported measurements.

The study has some limitations that should be taken into consideration. The use of a convenience sample and the sample size limit the generalization of the findings. The participants filled out their own surveys rather than by an interviewer. As with most dietary studies, this study relied on the dietary information provided by the participants: the frequency and amount of food consumption may not be precise. Additionally, the BFFQ may not represent food items the participants were consuming.

## 5. Conclusion

The study highlights the difference in the overweight and obesity rates among Filipino Americans when using the WHO Asian cut-off points compared to the International guideline. The continued use of the International guideline to determine obesity in the Filipino American population will mask the health risks in this population and delay primary prevention. It is necessary for the healthcare providers to utilize the WHO Asian recommendation for BMI and waist circumference cut-off points to improve prevention and provide early detection and treatment of cardiovascular and metabolic diseases.

The study also confirms an increased fat intake among Filipino Americans. With the increased prevalence rates of HTN and diabetes among the Filipino Americans [18–20, 31], there is a need for targeted interventions on improving dietary intake in this high-risk group. Future studies should include culturally competent interventions in improving dietary fat intake and decreasing obesity.

## Figures and Tables

**Table 1:** Dietary intake.

Participants	Caloric intake (kcal)	Carbohydrate intake (%)	Fat intake (%)
Men	1648.6	50.8	34.6
Women	1436.5	49.9	35.5
Total	1513.2	50.2	35.2

**Table 2:** Correlations between dietary intake, age, and immigration information.

Dietary intake	Age	Length of residency	Immigration age
Calories	−0.043 (0.536)	−0.124 (0.072)	0.050 (0.471)

Fat	−0.122 (0.78)	0.033 (0.639)	−1.158^*∗*^ (0.022)

Carbohydrate	0.212^*∗*^ (0.002)	−0.009 (0.892)	0.246^*∗*^ (<0.001)

^*∗*^Findings that reached statistical level. Significance established at *p* < 0.05.

**Table 3:** Comparison of BMI categories using the International guideline and the WHO Asian recommendation.

BMI category	International BMI guideline	WHO Asian BMI
*Overall*		
Normal (%)	54.3	25.2
Overweight (%)	36.2	49.5
Obese (%)	9.5	25.2

*Men*		
Normal (%)	38.2	13.2
Overweight (%)	46.1	55.3
Obese (%)	15.8	31.6

*Women*		
Normal (%)	63.4	32.1
Overweight (%)	30.6	46.3
Obese (%)	6	21.6

**Table 4:** Comparison of waist circumference categories using the International guideline and the WHO Asian recommendation.

Waist circumference category	International waist circumference cut-off	WHO Asian waist circumference cut-off
*Men*		
Normal (%)	80.3	50
Increased risk (%)	19.7	50

*Women*		
Normal (%)	76.1	49.3
Increased risk (%)	23.9	50.7

## Data Availability

The data used to support the findings of this study are available from the corresponding author upon request.
